# Plasmonic-multimode-interference-based logic circuit with simple phase adjustment

**DOI:** 10.1038/srep24546

**Published:** 2016-04-18

**Authors:** Masashi Ota, Asahi Sumimura, Masashi Fukuhara, Yuya Ishii, Mitsuo Fukuda

**Affiliations:** 1 Department of Electrical and Electronic Information Engineering, Toyohashi University of Technology, 1-1 Hibarigaoka, Tempaku-cho, Toyohashi-shi, Aichi 441-8580, Japan

## Abstract

All-optical logic circuits using surface plasmon polaritons have a potential for high-speed information processing with high-density integration beyond the diffraction limit of propagating light. However, a number of logic gates that can be cascaded is limited by complicated signal phase adjustment. In this study, we demonstrate a half-adder operation with simple phase adjustment using plasmonic multimode interference (MMI) devices, composed of dielectric stripes on a metal film, which can be fabricated by a complementary metal-oxide semiconductor (MOS)-compatible process. Also, simultaneous operations of XOR and AND gates are substantiated experimentally by combining 1 × 1 MMI based phase adjusters and 2 × 2 MMI based intensity modulators. An experimental on-off ratio of at least 4.3 dB is confirmed using scanning near-field optical microscopy. The proposed structure will contribute to high-density plasmonic circuits, fabricated by complementary MOS-compatible process or printing techniques.

Plasmonic logic devices are of interest because of their potential for high-speed information processing with high-density integration beyond the diffraction limit of light^1^,^2^,^3^. They may be used for subwavelength- or nanometer- scale optical computing with a higher effective refractive index by applying slot waveguides^3^,^4^,^5^. However, the number of plasmonic logic gates that can be cascaded and that use interference of multiple signals is limited by complicated signal phase adjustments. Here, we demonstrate a half-adder operation with simple phase adjustment using plasmonic multimode interference (MMI) devices, composed of dielectric stripes on a metal film, which can be fabricated by a complementary metal-oxide semiconductor (MOS)-compatible process. We simply adjusted the phase shift of the plasmonic signal by designing fundamental-mode effective waveguide widths of logic gates to interfere with plasmonic signals in the MMI devices. The proposed device can process multiple plasmonic logic operations simultaneously without the use of an MOS field-effect transistor.

Surface plasmon polaritons (SPPs) — collective oscillations of free electrons at a metallic surface — can confine optical signals into dielectric stripes beyond the diffraction limit of propagating light. Logic gates using SPPs have been reported using nanometer-scale Ag wires^1^, polyvinyl alcohol stripes^2^, and Ag–air–Ag slot waveguides^3^. These devices are composed of single-mode waveguides and use interference for logical operations. With such single-mode-interference-based logic devices, it is difficult to process multiple logic operations and splitting signals into multiple outputs with a simple structure. In addition, they require phase adjustment with use of modulators^6^,^7^, bending waveguides^8^,^9^ and resonators^10^,^11^. Although they are useful for phase adjustment and flexible wiring, frequent use of these structures will contribute to complex patterns of plasmonic circuits.

MMI is widely applied for beam splitting^12^,^13^,^14^, combining signals^15^,^16^ and switching^17^,^18^,^19^, because of advantages such as a low insertion loss and simple fabrication. Plasmonic MMI devices, composed of dielectric multimode waveguides on a metal film, can be used to realize interference-based SPP computing and optional phase shift adjustment in a simple structure. They are designed by determining the propagation coefficient of waveguides based on self-imaging^20^, and the propagation properties of multimode waveguides which reproduce single or multiple images of the input-field distribution periodically.

In this Letter, we propose a MMI based plasmonic logic circuit, which is composed of SiO_2_ stripes on a Au film. Half-adder operations were demonstrated numerically and experimentally, by combining plasmonic MMI with XOR and AND gates with simple phase adjusted structures.

## Results

**Figure 1:**
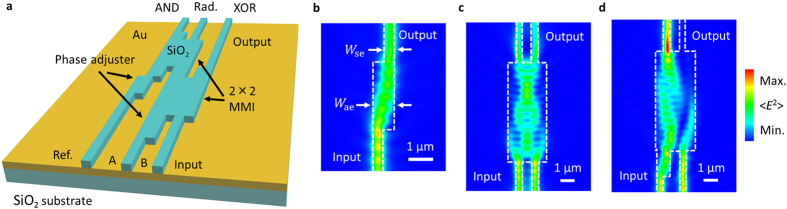
Plasmonic logic circuit using the MMI structure. (**a**) Schematic illustration of an MMI-based plasmonic half adder, composed of 1 × 1 MMI phase adjusters and 2 × 2 MMI intensity modulators. Radiation output, which suppresses radiation loss caused by opposite phase interference in the case of single-mode-waveguide-based logic, is abbreviated as Rad. (**b**) Numerical simulation results of the optical field distribution on a plasmonic phase adjuster. Here, the width, height, and length of the phase adjuster were 900 nm, 500 nm and 1900 nm, respectively, and the width of the single-mode waveguide was 400 nm. (**c**) Numerical simulation results of the optical field distribution at the 2 × 2 MMI intensity modulator, where the plasmonic signals were input with no phase difference. (**d**) Optical field distribution when the input phase difference was set to π/2. The width and length of the 2 × 2 MMI intensity modulator were set to 2800 nm and 7000 nm, respectively. These structures have the potential to be further minimized using gap plasmons.

In Fig. 1a, a schematic illustration is shown of the proposed half adder, which combines 1 × 1 MMI phase adjusters (Fig. 1b) and a 2 × 2 MMI intensity modulator (Fig. 1c,d). In the proposed plasmonic circuit, signals propagate along SiO_2_ stripes with a plasmonic velocity which depends on the effective refractive index, although there is a degree of signal dispersion throughout the MMI structures. The proposed device has two inputs and one reference, where the reference signal intensity was set at 25% of the input signal intensity. In the 2 × 2 MMI intensity modulator, each input plasmon forms an image for each output; hence, the phase difference between the two output signals is π/2^20^. In the 1 × 1 MMI phase adjuster set at the input A and the reference (abbreviated as Ref. in Fig. 1a), the diffracted input SPPs were efficiently coupled to an output waveguide. These structures can be designed to include another optional phase difference between the inputs. By combining these MMI structures, the 2 × 2 MMI intensity modulator is used to obtain an output with an optimal signal intensity, which results from the interference of two inputs with an optimal phase difference.

A truth table and the on-off ratios of the proposed half-adder operations, which combine XOR and AND logic, are shown in Tables 1 and 2, respectively. To carry out the XOR operation, input SPPs should interfere with antiphase SPPs at the XOR output. However, when two SPPs are input with coordinate phases, the signals interfere at each output with a phase difference of π/2 as shown in Fig. 1c. We controlled the phase difference between the two inputs by designing a 1 × 1 MMI phase adjuster, set at input A. By adjusting the phase difference between the two inputs to be π/2, images were formed at each output of the MMI structure, and interfered with the antiphase waves at the XOR output. An additional output, at which the images produced interference with the coordinate phase waves, acted as a multiplexing output, as shown in Fig. 1d. This output contributes to the AND operation which is performed at a second 2 × 2 MMI structure. As mentioned above, a 2 × 2 MMI structure produces two outputs, at which the two input signals and the reference signal interfere with an antiphase or coordinate phase signal. Here, the reference signal interferes with the multiplexing output of the previous MMI, and therefore an on-off ratio of about 10 dB can be obtained at the AND output. The phase difference between the reference and multiplexing signal is also adjusted to be π/2, where the multiplexing signal lags behind the reference phase. An additional output in the second 2 × 2 MMI acts as a radiation port, which suppresses radiation loss caused by opposite phase interference in the case of single-mode-waveguide-based logic.

The phase adjusters consisted of 1 × 1 mirror-imaged MMI structures, in which the input and output were located asymmetrically on the lateral left and right side, are shown in Fig. 1b. The phase shift in the MMI structures can be directly calculated using the self-imaging property. In the proposed device, because of propagation losses of SPPs, the structures of the phase adjusters should be compact to achieve low insertion loss. Then, according to the self-imaging property, the MMI length of the adjuster was determined from the MMI widths based on the number of guided modes. Therefore, the MMI width was determined within a narrow range, which included the fundamental and first guided modes. A single-mode waveguide width of 400 nm and thickness of 500 nm were chosen because it offers relatively strong confinement of SPPs. The proposed structures were operated by a light with wavelength of 1310 nm in free space. In contrast to the case of large scale MMI structures, which include many guided modes, compact plasmonic MMI structures which support few guided modes and thus accumulate small deviations. Therefore, an MMI width of 900 nm, including the fundamental and first guided modes, were chosen for the compact π/2-phase shift. Here, the phase shift of the plasmonic signal at the adjuster, which has a variable lag Δ*φ*_a_, is approximately expressed by


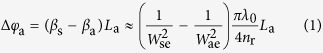


where *β*_s_ and *β*_a_ are the propagation coefficients of the fundamental modes, *W*_se_ and *W*_ae_ are the effective waveguide width of the fundamental modes of the single mode waveguide and phase adjuster, respectively, *L*_a_ is the length of the phase adjuster, *n*_r_ is the refractive index of the dielectric patterns, and *λ*_0_ is the free space wavelength of the incident light. In the design of the half adder, the plasmonic MMI structures should be compact to achieve a low insertion loss when considering the influence of propagation losses based on Joule heating of SPPs^21^,^22^, as mentioned above. Hence, plasmonic MMI waveguides have a narrow width which includes a few guided modes^23^,^24^,^25^, and the phase shift of the 1 × 1 MMI and 2 × 2 MMI are easily determined by the waveguide widths.

**Figure 2:**
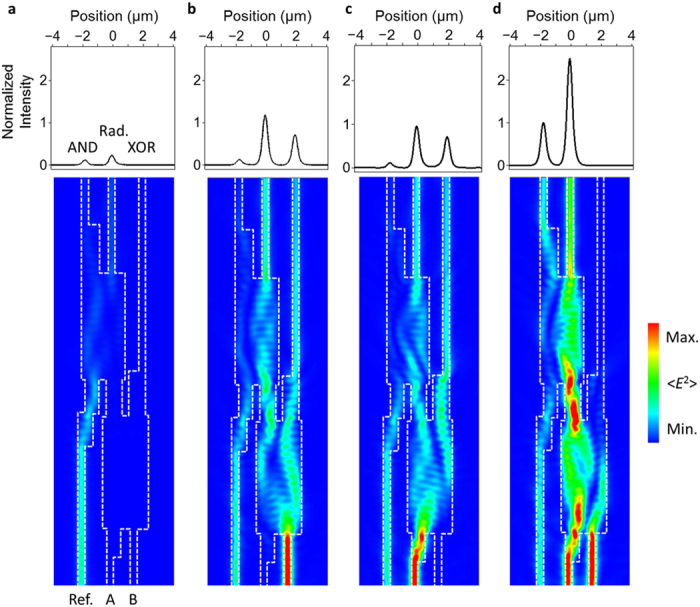
Simulated optical intensity distributions of the proposed half adder. (**a**–**d**) Optical distributions and output characteristics of “00”, “01”, “10”, “11” input states. Plasmonic signals interfered through the MMI structures, and half-adder operations were confirmed numerically. Upper parts of panels (**a–d**) are the output characteristics averaged over 1 μm and normalized with respect to the maximum value of AND “11” output.

We numerically simulated the SPP fields in the MMI patterns of the half adder and calculated the on-off ratio for the XOR and AND output using a 3D finite-difference time-domain (FDTD) method with electromagnetic wave analysis software (Fujitsu, Poynting for Optics). The refractive indices of SiO_2_ and Au were set at 1.447 and 0.41 + 8.37*i*, respectively^26^,^27^. In the SiO_2_ single-mode waveguides, the effective wavelength of SPPs (1063 nm) is close to the wavelength of propagating light (1068 nm). Plasmonic signals can therefore propagate along SiO_2_ patterns with approximate light velocity. The simulated optical intensity distributions of MMI for half-adder operations are shown in Fig. 2a–d. From these numerical results, an on-off ratio of at least 7.43 dB was confirmed, as shown in Tables 1 and 2. In Fig. 2, to carry out the XOR operation, input SPPs interfere with antiphase SPPs at the XOR output as mentioned above, and the XOR output signal therefore has input-dependent phase change π. To carry out the AND operation, the input SPPs interfere with the reference SPPs, and the AND output signal does not change the phase because of the input multiplexing in phase. Hence, the AND output can be connected in a cascaded circuit, although the XOR output cannot be connected to the other logic gates due to the input-dependent phase change. In the proposed half adder, the XOR and AND operation gives a sum and carry output, respectively. If the proposed half adder is used in a cascaded multi-bit adder, only the AND output will be connected to the upper bit adder and the XOR output will be detected as an intensity signal of the sum output.

**Figure 3:**
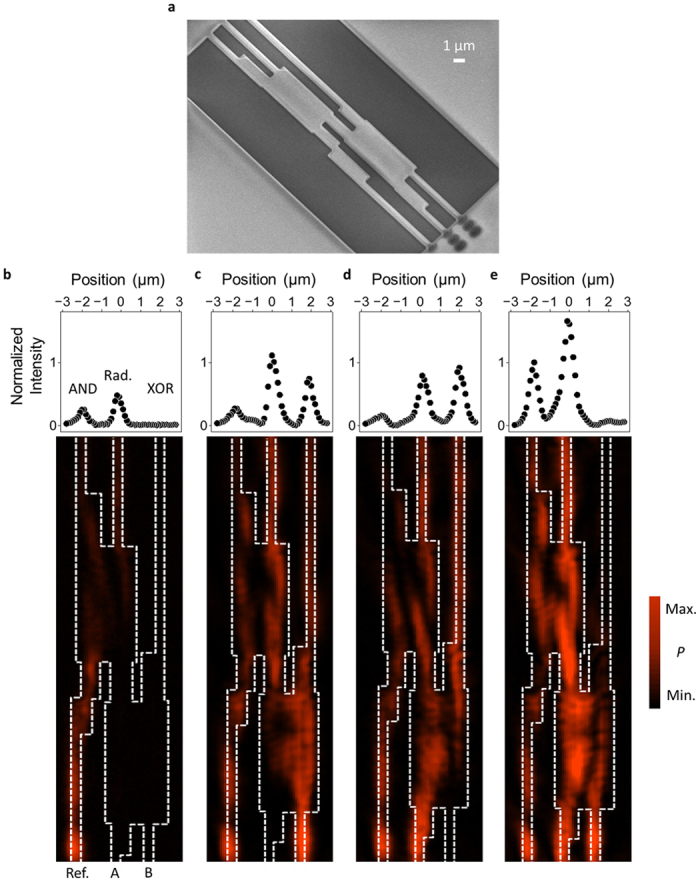
Experimental results of the fabricated devices. (**a**) Scanning electron micrographs of the proposed device, which was fabricated using focused ion beam etching. (**b**–**e**) Observed near-field optical images and output characteristics of the half adder using the MMI structure. Upper parts of panels (**b**–**e**) are the output characteristics averaged over the 1 μm and normalized with respect to the maximum value of the AND “11” output.

Scanning electron micrographs are shown in Fig. 3a of the devices fabricated based on the FDTD simulations. The devices were composed of 500-nm-thick SiO_2_ patterns on a 550-nm-thick Au film, which was deposited on a SiO_2_ substrate and formed by focused ion beam etching. Here, the width of the fabricated single-mode waveguide, phase adjuster, and MMI intensity modulator were 380 nm, 890 nm and 2800 nm, respectively. The length of the fabricated phase adjuster and MMI intensity modulator were 3060 nm and 6490 nm. SPPs were generated via the gratings using the manner similar to that reported in ref. 23, and the number of input SPPs was determined by comparing the results with and without the grating. In Fig. 3b–e, we show the distribution of the near-field optical intensity in the plasmonic MMI structure, which was observed with a scanning near-field optical microscope. The near-field optical patterns corresponding to the input, reference, and output waveguides were clearly observed with an on-off ratio of at least 4.3 dB, as shown in Table 2 and the upper parts of panels (b–e) in Fig. 3. When compared with the simulated results, the experimental on-off ratios were lower owing to the imprecise fabrication process and positioning accuracy of the grating. This lower on/off ratio can be improved by improving fabrication accuracy or connecting the outputs of the device to a plasmonic equalizer which use plasmonic amplification^28^. The results confirm the feasibility of logic operations in simple plasmonic MMI structures.

We have demonstrated optical half-adder operations using the plasmonic MMI effect. The proposed device, which was composed of SiO_2_ stripes on a Au film, could be operated at high speed and with a large capacity for information processing based on intensity modulation without use of an MOS field-effect transistor. The half adder, one of the main devices for information processing using Boolean logic, is composed of XOR and AND gates. The simultaneous operations of XOR and AND gates were substantiated experimentally by combining 1 × 1 MMI based phase adjusters and 2 × 2 MMI based intensity modulators. In the phase adjusters, we controlled the phase shift of the plasmonic signal by determining the propagation coefficients of the fundamental guided modes of the waveguides. An experimental on-off ratio of at least 4.3 dB was determined using scanning near-field optical microscopy. We believe that this work will contribute to high-density plasmonic circuits, fabricated by complementary MOS-compatible process or printing techniques.

## Methods

The experiments were performed using a scanning near-field optical microscope (SNOM, Japan Spectroscopic Company, NFX-520). The back side of a SiO_2_ substrate was irradiated with collimated incident light, which was chopped at a frequency of 270 Hz. Plasmonic signals were generated via the gratings, and the incident light from a tunable laser output (Koshin Kogaku, LS-601A) was spotted using a collimated fiber (Go Foton, C-OPCL-SMF-103/FC).

For the SNOM, plasmonic MMI patterns were detected with a photo-multiplier tube (Hamamatsu Photonics, H10330B-75) using near-field optical probe scanning at the surface of the device. The probe was controlled by an XYZ piezoelectric stage (Nano Control, A118-01) and controller (Nano Control, NCM7302C). The photo-multiplier output was connected to a digital lock-in amplifier (NF Corporation, LI5640), the output from which was monitored by the SNOM system and used to analyze the chopped plasmonic MMI patterns.

## Additional Information

**How to cite this article**: Ota, M. *et al.* Plasmonic-multimode-interference-based logic circuit with simple phase adjustment. *Sci. Rep.* **6**, 24546; doi: 10.1038/srep24546 (2016).

## Figures and Tables

**Table 1:** Truth table of half-adder operations consisting of combinations of XOR and AND operations in the proposed device.

Input Power			Logical Output		Numerical Output		Experimental Output	
A	B	Ref.	XOR	AND	XOR	AND	XOR	AND
0	0	0.25	0	0	0.00	0.12	0.02 ± 0.02	0.24 ± 0.04
0	1	0.25	1	0	0.72	0.13	0.72 ± 0.03	0.27 ± 0.05
1	0	0.25	1	0	0.71	0.11	0.99 ± 0.09	0.16 ± 0.05
1	1	0.25	0	1	0.01	1.00	0.09 ± 0.02	1.00 ± 0.09

Numerical and experimental outputs are normalized with respect to the maximum value of AND “11” output.

**Table 2:** On-off ratio of half-adder operations using the plasmonic MMI.

Logic states	Numerical On-Off ratio (dB)	Experimental On-Off ratio (dB)
AND (MAX)	9.65	5.82
AND (MIN)	8.90	5.35
XOR (MAX)	26.19	17.16
XOR (MIN)	21.82	8.97
Entire half adder (MAX)	27.64	17.16
Entire half adder (MIN)	7.43	4.30
